# Food security: the challenge of increasing wheat yield and the importance of not compromising food safety

**DOI:** 10.1111/aab.12108

**Published:** 2014-02-21

**Authors:** T Curtis, N G Halford

**Affiliations:** 1 Plant Biology and Crop Science Department, Rothamsted ResearchHarpenden, Hertfordshirex, UK

**Keywords:** acrylamide, climate change, crop biotechnology, genetics, plant breeding, processing contaminants, quantitative trait loci, *Triticum aestivum*

## Abstract

Current wheat yield and consumption is considered in the context of the historical development of wheat, from early domestication through to modern plant breeding, the Green Revolution and wheat’s place as one of the world’s most productive and important crops in the 21st Century. The need for further improvement in the yield potential of wheat in order to meet current and impending challenges is discussed, including rising consumption and the demand for grain for fuel as well as food. Research on the complex genetics underlying wheat yield is described, including the identification of quantitative trait loci and individual genes, and the prospects of biotechnology playing a role in wheat improvement in the future are discussed. The challenge of preparing wheat to meet the problems of drought, high temperature and increasing carbon dioxide concentration that are anticipated to come about as a result of climate change is also reviewed. Wheat yield must be increased while not compromising food safety, and the emerging problem of processing contaminants is reviewed, focussing in particular on acrylamide, a contaminant that forms from free asparagine and reducing sugars during high temperature cooking and processing. Wheat breeders are strongly encouraged to consider the contaminant issue when breeding for yield.

## Introduction

The domestication of wheat approximately 10 000 years ago in the Fertile Crescent was perhaps the most important step in humankind’s transition from hunter-gatherer and nomadic herder to sedentary farmer. The earliest cultivated forms were diploid (genome AA) (einkorn) and tetraploid (genome AABB) (emmer) species that originated from the south-eastern part of Turkey (Shewry, 2009). Wheat changed a great deal over the subsequent millennia, as farmers selected the best seed from one year’s harvest to sow for the next year. One obvious difference was that the grain became much larger, but Shewry (2009) highlights two other key changes associated with domestication: the loss of shattering of the spike at maturity, a trait that ensures seed dispersal in the wild but causes seed loss in agriculture, and the change from hulled forms, in which the glumes (bracts enclosing the grain, which are removed as chaff on threshing) are stuck tightly to the seed, to free-threshing forms. Another development that occurred only within agriculture was the hybridisation of tetraploid wheat with a wild grass, *Triticum tauschii* (goat grass; also called *Aegilops tauschii* and *Aegilops squarosa*) to give a hexaploid, genome AABBDD. Today, about 95% of cultivated wheat is hexaploid, with the other 5% being tetraploid durum wheat grown in the Mediterranean region and used mostly for pasta (Shewry, 2009).

Wheat cultivation enabled enough food to be produced to support cities for the first time and led to the rise of the great Middle Eastern empires of Babylon, Assyria and Egypt. Wheat cultivation also transformed people’s lives in more northerly latitudes, where the ability to store the grain for long periods was invaluable in surviving the European winter. As a result, wheat can be regarded as the foundation stone on which western civilization was built. Wheat is now grown more widely than any other crop and a plethora of uses have been developed, including leavened bread, flat and steamed breads, biscuits, cakes, pasta, noodles, couscous and beer.

The 20th Century saw great improvements in many crops, with scientific plant breeding taking over from simple selection on the farm (reviewed by Halford, 2006). The principles of the new science were based on Mendel’s laws of inheritance, first published by the Association for Natural Research in 1866, under the title ‘Versuche über Pflanzen-Hybride’, effectively lost, then rediscovered in 1900. Scientific plant breeding, together with the development and use of fertilisers, pesticides and herbicides, had a dramatic effect on wheat yield. This came just in time to prevent a human disaster that was brewing in Asia by the 1960s, with the large populations of India and Pakistan, in particular, facing the prospect of widespread famine (Evans, 1998). The disaster was averted through the actions of Norman Borlaug, an American scientist who had been working at the Centro Internacional de Mejoramiento de Maíz y Trigo (CIMMYT) (International Centre for the Improvement of Maize and Wheat) in Mexico. Borlaug had developed disease-resistant, semi-dwarf wheat varieties that partitioned more assimilate to the grain, and led the introduction of these high-yielding varieties to India and Pakistan, resulting in an astonishing doubling of yield in a 5-year period between 1965 and 1970 (Evans, 1998). Similar improvements followed in rice, and in 1968 the US Agency for International Development (USAID) director, William Gaud, coined the phrase ‘Green Revolution’ to describe Borlaug’s achievements. A retrospective on Borlaug’s life and achievements has been written by his colleague, Christopher Dowswell (2009).

**Figure 1:**
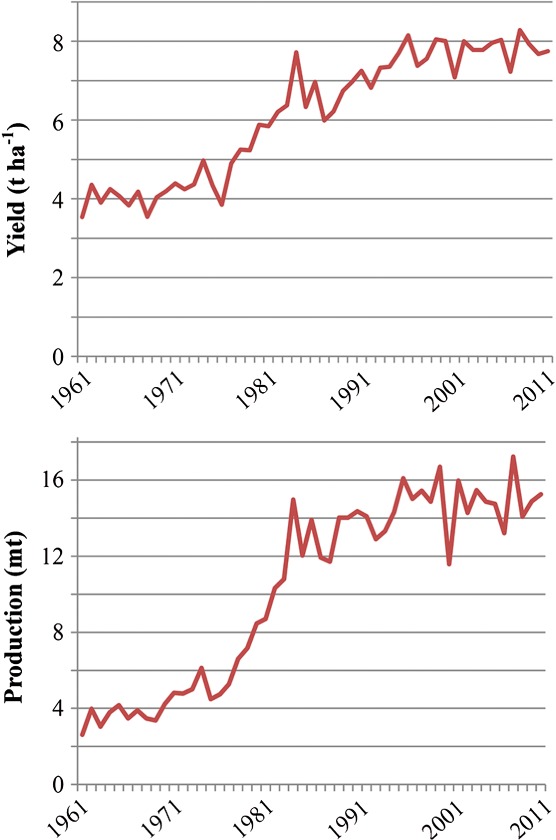
UK wheat yield (top) and production (bottom) from 1961 to 2011. Data from United Nations Food and Agriculture Organisation (2013).

Wheat yield continued to increase and through much of the last century wheat was the most-produced crop in the world. By 2010, world production of wheat grain was 651 mt, but yield in some regions had begun to level off. Fig. 1, for example, shows yield and production for UK wheat from 1961 to 2011 (Food and Agriculture Organisation of the United Nations, 2013). Yield rose sharply after the Second World War with food shortages providing a powerful incentive for improvement. In 1961 it was 3.5 t ha^−1^, and in just over two decades it more than doubled to reach 7.7 t ha^−1^ by 1984. However, there has been little improvement in more than a quarter of a century since then, with yield in 2011 actually being slightly lower at 7.6 t ha^−1^. Production followed a similar curve, but the increase was even greater, from 2.6 mt in 1961 to 15.3 mt in 2011 (Fig. 1). This was driven in part by the European Union’s Common Agricultural Policy, which provided generous subsidies for wheat production, resulting in many UK farmers switching from barley to wheat. The area of wheat harvested in the UK in 1961 was only 0.74 million ha, compared with 1.97 million ha in 2011.

Partly as a result of the plateauing in yield improvement in some regions, wheat has been overtaken in terms of tonnage by maize (844 mt) and rice (672 mt) (Food and Agriculture Organisation of the United Nations, 2013). Nevertheless, wheat grain remains the most traded crop commodity on world markets, the leading crop source of protein and, along with rice, the most important crop product for human consumption (more maize is used for animal feed and, in recent years, fuel production).

Table 1 shows the United Nation’s Food and Agriculture Organisation list of the world’s major wheat producers, each producing at least a million tonnes of grain in 2012. The European Union (EU) (with 28 Member States) tops the list at over 132 mt, followed by China, India, the USA and Russia. The area of land from which this is produced is also shown, as is the yield in tonnes per hectare. Yield varies greatly from country to country, and presents a few surprises. Globally, the average yield is 3.03 t ha^−1^. The average yield in the EU is higher, at 5.19 t ha^−1^, but even within the EU there is considerable variation, from over 8 t ha^−1^ in the Netherlands and Belgium to less than 2 t ha^−1^ in the severely water-limited conditions of Portugal and Cyprus (Table 2). Outside Europe, the yield in Egypt, for example, is higher than might be expected at 6.3 t ha^−1^, while that in the USA is only 3.12 t ha^−1^ and in Australia just 1.65 t ha^−1^. The highest recorded yields are much higher, with 13.99 t ha^−1^ obtained on a farm in Scotland, UK, in 1981, holding the record from that time until it was beaten by a figure of 15.64 t ha^−1^ obtained on a New Zealand farm in 2010 (Guinness World Records, 2013).

**Table 1:** Wheat production figures for countries producing more than a million tonnes of wheat in 2012, and global production figures (Food and Agriculture Organisation of the United Nations, 2013)

Country	Production (1000 tonnes)	Area (million ha)	Yield (tonnes per ha)
European Union	132 251	25.50	5.19
China	120 600	24.14	5.00
India	94 880	29.69	3.20
United States	61 755	19.82	3.12
Russian Federation	37 717	21.30	1.77
Canada	27 200	9.50	2.86
Pakistan	23 300	8.66	2.69
Australia	22 000	13.30	1.65
Ukraine	15 761	5.63	2.80
Turkey	15 500	7.80	1.99
Iran	14 000	7.00	2.00
Argentina	11 000	3.70	2.97
Kazakhstan	11 000	12.40	0.89
Egypt	8 500	1.35	6.30
Uzbekistan	6 700	1.40	4.79
Brazil	4 300	1.90	2.26
Afghanistan	4 150	2.51	1.65
Syria	3 700	1.60	2.31
Algeria	3 400	2.00	1.70
Morocco	3 400	3.14	1.08
Mexico	3 230	0.57	5.67
Ethiopia	3 100	1.50	2.07
Iraq	2 100	1.25	1.68
Belarus	2 100	0.60	3.50
Azerbaijan	2 000	0.69	2.90
Serbia	1 900	0.48	3.96
South Africa	1 900	0.51	3.72
Nepal	1 746	0.77	2.27
Uruguay	1 575	0.44	3.58
Tunisia	1 450	0.80	1.81
Chile	1 400	0.25	5.60
Paraguay	1 300	0.50	2.60
Turkmenistan	1 200	0.85	1.41
Bangladesh	1 100	0.42	2.62
Saudi Arabia	1 000	0.20	5.00
New Zealand	488	8.92	
Global	651 000	215.92	3.03

**Table 2:** Wheat production figures for European Union Member States for 2012 (Food and Agriculture Organisation of the United Nations, 2013)

Country	Production (1000 tonnes)	Area (million ha)	Yield (tonnes per ha)
France	40 301	5.30	7.60
Germany	22 432	3.06	7.33
United Kingdom	13 261	1.99	6.66
Poland	8 610	2.09	4.12
Italy	7 430	1.85	4.02
Romania	5 298	1.99	2.66
Spain	4 650	1.76	2.64
Denmark	4 525	0.61	7.36
Bulgaria	4 330	1.09	3.97
Hungary	3 740	1.06	3.52
Czech Republic	3 519	0.81	4.32
Lithuania	2 999	0.63	4.78
Sweden	2 289	0.37	6.24
Belgium	1 636	0.20	8.30
Greece	1 620	0.53	3.06
Latvia	1 540	0.35	4.34
Slovakia	1 365	0.63	4.01
Netherlands	1 302	0.15	8.59
Austria	1 275	0.31	4.14
Croatia	1 000	0.19	5.35
Finland	887	0.23	3.90
Ireland	618	0.10	6.31
Estonia	485	0.12	3.90
Slovenia	188	0.035	5.44
Luxembourg	79	0.015	5.46
Portugal	50	0.038	1.32
Cyprus	19	0.011	1.75
Malta	16	0.0028	5.71

The yield in the USA reflects wheat’s status in that country as a relatively low value crop that is grown under low intensity management. It contrasts with the average yield for maize in the same country, which is over 10 t ha^−1^ (Food and Agriculture Organisation of the United Nations, 2013). The yield in Australia is affected by low water availability and phosphate-deficient soils, and, despite its low yield, Australia is a major producer and exporter of wheat, thanks to the huge land area it has available and its relatively small population.

Understanding the underlying causes of the wide differences in yield figures in Tables 1 and 2 and how they could be addressed is key to increasing global wheat production. Arguably, the most effective strategy would be to increase yields in those countries or regions that have large areas of land available and where yields are currently low. Kazakhstan is an example; with a land area approximately equal to that of Western Europe and a population of only 16.5 million people, it is an important wheat producer and exporter, with 12.4 m ha of land used for wheat cultivation. However, its annual production in recent years has fluctuated widely, from 17.1 mt in 2009 to 9.6 mt in 2010, 22.7 mt in 2011 (a record year in the post-Soviet era) and back down to 11 mt in 2012. Wheat production in Kazakhstan is severely water-limited and the soil in some regions is affected by high salt concentrations and soil degradation (Gupta *et al.*, 2009). Extreme cold in winter and heat in summer are also problems. Yield in 2012 was only 0.89 t ha^−1^, the lowest of all the major producers. The export of surplus grain is also hampered by infrastructure and transport problems. These issues could be addressed, at least in part, through irrigation (currently there is very little), soil remediation and provision of a transport infrastructure to enable farmers to get their surplus grain onto the world market. Raising Kazakhstan’s yield to the global average of 3 t ha^−1^ would increase production by 26 mt compared with 2012, equivalent to 4% of global production.

## Challenges

World agriculture faces serious challenges if it is to meet demand in the coming decades and the first of these is that total consumption is rising. Table 3 shows the countries that consume most wheat, with consumption figures for 2012 and for 1962, half a century earlier (Food and Agriculture Organisation of the United Nations, 2013). In China, consumption increased more than sixfold in that half century, from just over 19 mt to 123 mt, overtaking the EU. Consumption in the EU has been fairly flat in recent years, the figure of 121.5 mt in 2012 representing an increase of only 7% over the figure in 1999. Note that the population of the EU block is approximately 504 million, while that of China is 1.35 billion, so *per capita* consumption of wheat is approximately 2.7 times higher in the EU than in China. The Chinese, however, consumed 144 mt of rice and 210 mt of maize in 2012, compared with 3.4 and 66 mt, respectively, in the EU.

**Table 3:** Changes in wheat consumption for selected countries from 1962–2012. Compiled using data from the United States Department of Agriculture

Country	Consumption 1962 (1000 tonnes)	Consumption 2012 (1000 tonnes)	Ratio 2012: 1962
Indonesia	24	6 450	269
Bangladesh	40	4 200	105
Thailand	32	1 950	60.9
Nigeria	87	3 290	37.8
Vietnam	(1963) 108	2 450	22.7
Sudan	156	2 280	14.6
Philippines	390	3 400	8.72
China	19 268	123 000	6.38
Morocco	1 352	8 400	6.21
Ethiopia	651	3 745	5.75
India	14 822	84 540	5.70
Egypt	3 308	18 400	5.56
Iraq	1 079	5 800	5.38
Tunisia	553	2 950	5.33
Iran	3 030	15 700	5.18
Korea	1 077	5 500	5.11
Algeria	1 778	9 050	5.09
Mexico	1 435	6 700	4.67
Brazil	2 432	11 000	4.52
Pakistan	5 378	23 200	4.31
South Africa	946	3 250	3.44
Australia	2 053	6 840	3.33
Argentina	3 643	6 000	2.92
Canada	3 756	9 350	2.49
Turkey	7 345	17 500	2.38
USA	16 305	38 109	2.34
Uzbekistan	3 975	7 700	1.94
EU15	48 881	(1998) 88 135	1.80
EU28	(1999) 113 228	121 500	1.07
USSR	53 915	(1986) 96 498	1.63
Russia	(1987) 50 981	34 000	0.67
Japan	4 390	6 700	1.53
Ukraine	(1987) 21 329	12 000	0.56

The increase in wheat consumption has been even more spectacular in other Asian countries, with Indonesia topping the table with a 269-fold increase between 1962 and 2012, followed by Bangladesh (105-fold), Thailand (60.9-fold) and Vietnam (22.7-fold). There was also a 37.8-fold increase in Nigeria and 14.6-fold increase in the Sudan. In contrast, consumption is actually falling in two of the countries on the list: Russia (a one third decrease since the break-up of the USSR in 1987), and the Ukraine (a 44% decrease).

The major consumers other than China and the EU in 2012 were India (84.5 mt), USA (38.1 mt), Pakistan (23.2 mt), Egypt (18.4 mt), Turkey (17.5 mt), Iran (15.7 mt) and Brazil (11 mt). Note that the figure for the USA is less than a third that of the EU. Maize consumption in the USA, however, was 265 mt in 2012. The USA is the leading wheat exporter, selling 29.9 mt for export in 2012, followed by the EU (19.5 mt), Canada (18.5 mt), Australia (16.5 mt), Russia (10.5 mt), Ukraine (6.5 mt), Kazakhstan (6.5 mt), Argentina (5 mt) and Turkey (3.3 mt). Note that consumption and export figures do not necessarily add up to total production because countries that export grain may also import grain of a different quality or end use.

**Figure 2:**
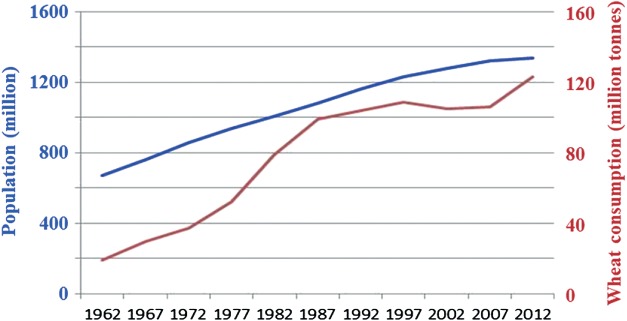
Growth in population and wheat consumption in China from 1962 to 2012. Annual *per capita* consumption was 29 kg in 1962 and 92 kg in 2012. Data: Food and Agriculture Organisation of the United Nations (2013).

Demand is rising partly as a result of population growth (world population more than doubled from 3.15 billion in 1962 to 7.05 billion in 2012 (Population Division of the Department of Economic and Social Affairs of the United Nations Secretariat, 2012)), but also because of increasing consumption *per capita*. In China, for example, while population doubled between 1962 and 2012, wheat consumption increased sixfold, from just under 20 to 123 mt, so *per capita* consumption trebled from 29 to 92 kg (Fig. 2). This has been driven by improving prosperity and a desire and increasing ability to pay for more meat (it requires much more grain to rear an animal for consumption than to feed people directly) (Popkin, 2006). Starchy grain crops are also being used increasingly for bioethanol production. In the USA, for example, 116 mt of maize grain was used for bioethanol production in 2012, representing 42.4% of total US maize grain production (United States Department of Agriculture Economic Research Service, 2013). This has had a knock-on effect on all crop commodity prices and in 2012 the UN Food and Agriculture Organisation (FAO) called on the US government to suspend the ‘ethanol mandate’, the stipulation in the 2005 US Energy Policy Act that 7.5 million gallons of bioethanol be produced annually by 2012. So far the US government has refused to do so.

Wheat starch is equally suitable for ethanol production, and three large facilities that have already opened or are in development in the UK, for example, will have the capacity between them to use 2.8 mt of wheat grain per annum, representing 19% of the UK’s average wheat harvest. As in the US, the development of this industry is to a great extent politically driven, with the EU’s 2009 Renewable Energy Directive committing all Member States to achieving a target of 10% of transport fuel coming from renewable sources by 2020. The dependence of the industry on political support is clear from the fact that one of the plants that has been built for bioethanol production from wheat grain in the UK is currently mothballed, with slowness in complying with the EU directive cited as one of the factors to blame, along with competition from imported ethanol. Nevertheless, bioethanol production is likely to have a rapidly increasing impact on wheat grain supply and cost as the industry picks up.

**Figure 3:**
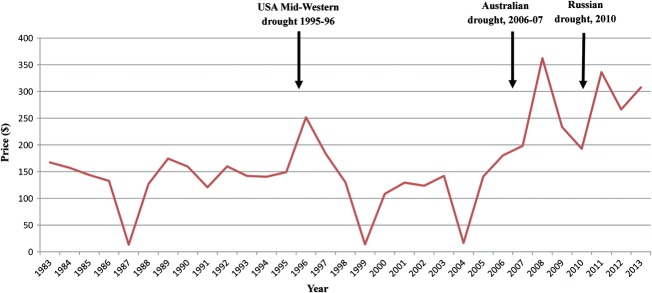
US hard red wheat trading price from 1983 to 2013. Data: United States Department of Agriculture. The timings of extreme weather events affecting major wheat exporting countries are indicated.

The rise in demand for wheat has led to a steady increase in its price on world markets over the last decade, as shown in the graph of the US hard red wheat trading price in Fig. 3. The price has also been affected by severe droughts in the USA in 1995–1996, Australia in 2006–2007 and Russia in 2010. These countries are important wheat exporters and the loss of production caused the wheat price to spike at $250 per tonne in 1996, $350 per tonne in 2008 and $330 per tonne in 2011, compared with its trend price from 1983 to 2005 of around $150 per tonne (Fig. 3). Prices on the London International Financial Futures and Options Exchange (LIFFE) followed a similar pattern, hitting a record of £200 per tonne in December 2011. These prices have not fallen back: the US price is still over $300 per tonne, while the London LIFFE price was £193 per tonne for May 2013. The Russian drought of 2010 also led to more political intervention, with the Russian government banning wheat exports in order to preserve stocks for its own population to use, a salutary lesson for those who have argued that countries such as the UK that are not self-sufficient in food will always be able to import enough food to meet their needs.

The rise in demand for wheat is clearly going to continue: the median projection for world population is for it to rise from its current level of 7.20 billion to 9.55 billion by 2050 and 10.87 billion by 2100 (Population Division of the Department of Economic and Social Affairs of the United Nations Secretariat, 2012). At the same time, the ability to meet that demand may be compromised by a number of factors, including climate change, lack of sufficient fresh water, soil erosion, salination, pollution and the loss of agricultural land to other uses. The upward trend in prices seen over the last decade is therefore likely to continue, and arguably the era of cheap food and global food surpluses is already over and food security rather than artificially maintaining prices to keep farmers in business is at the top of the political agenda. Not necessarily a bad thing for farmers, of course, but worrying for consumers and for governments as rising food prices feed into inflation figures.

If the supply of wheat is to be secured, plant breeding will have to play its part, and several national and international programmes have been set up recently with the aim of achieving significant increases in wheat yield (for example, the G20-led Wheat Initiative: http://urgi.versailles.inra.fr/Species/Wheat/Projects2/Wheat-Initiative; the Wheat Yield consortium: http://www.cimmyt.org/en/newsletter/38-2009/461-wheatwarriors-the-struggle-to-break-the-yield-barrier (Reynolds *et al.*, 2011); 20:20 Wheat: http://www.rothamsted.ac.uk/Content.php?Section=Research&Page=Wheat). The following sections of this review describe recent advances in our knowledge and understanding of the genetics of wheat yield, and how that knowledge could be turned into tools for wheat breeders to meet the challenges of the coming decades. The potential of biotechnology in wheat breeding and the obstacles to its development are also considered and studies that predict the effects of global warming and identify the traits that will be important if wheat breeders are to be able to respond to climate change are described.

In the face of the challenges that we have discussed in this section, crop scientists and breeders could be excused for ignoring all other issues as they strive to boost wheat yields. We strongly advise against such a course of action because another issue has arisen in recent years that is just as important to the food industry: that is the problem of processing contaminants and their implications for food safety and regulatory compliance. A section of the review therefore focuses on the issue of processing contaminants, using acrylamide, which forms from free asparagine and reducing sugars at high temperatures, as an example, and the review concludes with advice to breeders not to ignore the issue of food safety.

## The complex genetics controlling yield

Yield in wheat is a complex, polygenic genetic trait, with multiple genes contributing to yield potential and many more to how much of that potential is realised when the crop is challenged by particular abiotic and biotic stresses. It is affected by the efficiency with which wheat plants harvest the energy of sunlight through photosynthesis, mainly in mature leaves (source leaves) and how much of the sucrose that is made by photosynthesis is exported from the source leaves for use by non-photosynthetic (carbon sink) organs, including the grain. It is almost certainly also affected by the sinks themselves, with unloading and utilisation of sucrose arriving at sink organs imposing a drag on sucrose in the phloem and affecting the partitioning of carbon between different organs. The proportion of total biomass (B) that is allocated to the grain is the harvest index (HI), so grain yield (Y) = B × HI.

HI was improved dramatically during the ‘Green Revolution’, with genes for reduced height being incorporated into breeding programmes all over the world (Austin *et al.*, 1980; Evans, 1998). These genes affected the biosynthetic pathway for a hormone, gibberellin (GA) (Hedden, 2003). As well as partitioning a greater proportion of assimilate and other resources to the grain, shorter plants were less susceptible to lodging, in which the stalk falls over in the wind and rots on the ground. Importantly, more of the nitrogen supplied in fertiliser to these new varieties would be partitioned to the grain rather than promoting more vegetative growth and making the plants more susceptible to lodging. As a result, yields increased dramatically while total biomass was reduced.

It is generally accepted that HI cannot be improved much further, because it is already close to 60% in modern elite varieties and, clearly, the plant needs leaves to photosynthesise, roots to acquire water and minerals, and stalks to support the leaves and heads. This assumption has not been tested as far as we are aware; however, it seems likely that further increases in yield will involve increases in biomass while HI remains the same, reversing the trend of the last few decades. This will require an increase in carbon fixation through photosynthesis, which in turn is dependent on radiation use efficiency (RUE_PAR_). This could be achieved by increasing either the photosynthetic area or the photosynthetic capacity (amount of carbon fixed per unit area) (Long *et al.* 2006; Murchie *et al.*, 2009; Parry & Hawkesford, 2010; Murchie & Niyogi, 2011; Parry *et al.*, 2011, 2013; Reynolds *et al.* 2012).

Hawkesford *et al.* (2013) considered the establishment of an efficient canopy to be a key to increased production. They argued that carbon fixation could be increased by optimising canopy architecture and light capture efficiency, improving photosynthetic efficiency and extending the grain-filling period. The canopy also plays a role in nitrogen assimilation and storage, and the remobilisation of nitrogen to the grain during crop maturation. The authors pointed out that any changes to canopy architecture must not compromise resistance to disease or lodging, and other stresses such as heat and drought could be added to that list. Murchie *et al.* (2009) and Reynolds *et al.* (2012) considered that canopy architecture might already be close to optimised, although they discussed the possibility of improving leaf posture, size or density and the consequences for light within the canopy. Zhu *et al.* (2012), on the other hand, pointed out that plant canopy architecture evolved in plants that were competing with their neighbours in a way that does not occur in agriculture, where crop plants are evenly spaced and weeds are controlled, and that there may be more scope for improvement as a result.

Foulkes *et al.* (2011) emphasised the importance of optimising resource partitioning to the grain in favour of the vegetative parts of the plant. This approach assumes that yield is sink- as well as source-limited in wheat, and would increase if more assimilate were partitioned to the developing spikes in favour of the vegetative parts of the plant. It must be born in mind when considering this that assimilate may be partitioned to the leaves but remobilised to the grain as the leaves senesce during late development. Nevertheless, the authors identified several sink-oriented strategies, including maximising spike fertility and grain number, optimizing spike growth to maximize grain number and HI, improving spike fertility by making florets more resilient to environmental stress, and improving potential grain size and filling. These would be combined with improved lodging resistance. The effect of spike size was also investigated by Gaju *et al.* (2009), using genotypes with a large-spike phenotype. One large-spike phenotype line did yield 9% more than the control in one of three seasons, but generally the large-spike phenotype was associated with a lower spike number per square metre and lower grain number per plant.

Reynolds *et al.* (2009), on the other hand, concluded that both source- and sink-oriented traits were important. For example, they considered that improving photosynthesis was important to increase yield potential, but that any improved yield potential could be realised only if spike fertility were improved. They took evidence of underutilised photosynthetic capacity to indicate that florets aborted unnecessarily, reducing grain number, and that better understanding of the physiological and genetic basis for this was necessary so that floret abortion could be minimised.

## Quantitative trait loci (QTL)

An alternative approach to identifying the limiting factors for yield and then manipulating them is to work backwards by comparing similar genotypes with a range of yields and identifying the parts of the genome or individual genes that underlie this variation. This approach makes no assumptions about what those genes might be or what areas of physiology or biochemistry they might affect. As well as being a complex trait that is affected by multiple genes, yield is a quantitative trait that varies continuously (in contrast to traits controlled by single genes, in which allelic variation within a gene may result in step-wise differences). In recent years, the study of complex traits has been greatly facilitated by the development of tools and resources to identify and characterise quantitative trait loci (QTL) (Snape *et al.*, 2007); that is stretches of a genome that contain or are linked to genes that affect the trait. Quantitative trait loci may contain many individual genes, but QTL identification can be the first step towards the characterisation of the relevant genes themselves, or the QTL may be used by breeders without knowing which genes within the QTL are responsible for the improvement they are aiming for.

It is now possible to identify QTL in wheat much more rapidly than in the past because of the availability of doubled haploid mapping populations. These are produced by crossing two genotypes and then inducing a haploid cell to undergo chromosome doubling. The haploid cell may be a cultured microspore or a haploid embryo resulting from chromosome elimination after wide crossing. The other pre-requisite for QTL analysis is the availability of detailed genetic maps for both parental genomes giving the relative position of molecular markers over the whole genome. Statistical analysis is then used to associate differences in the score for the trait of interest in the closely related individuals within the population with the presence of specific molecular markers. Allelic variation within the QTL may be distinguished through single nucleotide polymorphisms (SNPs) and insertion/deletion polymorphisms in polymerase chain reaction products spanning intron sequences.

The position of each gene may be inferred from rice, barley and *Brachypodium distachyon* genome data (International Rice Genome Sequencing Project, 2005; International Brachypodium Initiative, 2010; International Barley Genome Sequencing Consortium, 2012). Differences between members of multigene families and between different alleles can then be associated with differences in the trait of interest, allowing the development of genetic markers for use in marker assisted selection by breeders. Genome nucleotide sequence data is, of course, of huge benefit to these studies and the availability of annotated, assembled wheat genome data would represent another step forward for wheat breeders. Progress is rapid in this area and wheat genome data is now available (http://www.cerealsdb.uk.net) (Brenchley *et al.*, 2012; Wilkinson *et al.*, 2012; Winfield *et al.*, 2012). It is not yet assembled into chromosomes, but a draft assembly of gene-rich regions has been constructed.

Snape *et al.* (2007) used large-scale analysis of several mapping populations, grown over different environments and seasons, to identify QTL and genes responsible for yield and yield × environment variation in European winter wheat. The populations were grown at drought-prone sites with and without irrigation. The most significant genes under these conditions were associated with flag leaf senescence. Quantitative trait loci underlying variation for stem soluble carbohydrate reserves were identified, and this trait was positively correlated with yield under both irrigated and non-irrigated conditions. Analyses of the populations grown at sites in the UK, France and Germany identified some QTL for yield that were significant over multiple sites and seasons, and some that were specific to a season or a site.

Another study to identify QTL for yield and yield components (1000 grain weight, HI, average seed weight per spike and spike number per square metre) was undertaken by Cuthbert *et al.* (2008) using a doubled haploid population of 402 lines developed from a cross between spring wheat varieties Superb (high yielding) and BW278 (low yielding). Five grain yield QTL were detected, on chromosomes 1A, 2D, 3B and 5A, showing a combined increase in yield of 34.4% over the population mean. Two QTL for grain yield were also identified on chromosome 3B in a study by Bennett *et al.* (2012). These QTL were detectable under drought and heat stress as well as optimal conditions, accounting for 22% of the variance.

Wu *et al.* (2012) identified QTL affecting grain yield both directly and indirectly using doubled haploid lines derived from a cross between two Chinese varieties, Hanxuan 10 and Lumai 14. They considered 10 traits that are associated with yield: grain yield per plant, number of spikes per plant, number of grains per spike, 1000 grain weight, total number of spikelets per spike, number of sterile spikelets per spike, proportion of fertile spikelets per spike, spike length, density of spikelets per spike and plant height, across up to 23 year × location × water regime environments in China. Twelve QTL for yield per plant were identified, spread across all chromosomes except 6D. They included three QTL described as ‘striking’ by the authors on chromosomes 1B, 2B and 4B. Progeny derived by selecting for 1000 grain weight, number of grains per spike and higher grain yield were found to have an increased frequency of QTL for the three selected traits and also for the number of spikelets per spike, proportion of fertile spikelets per spike, spike length and plant height, and a lower frequency of QTL for the number of sterile spikelets per spike. The authors concluded that it should be feasible to breed high-yielding wheat using marker-assisted selection.

Yuan *et al.* (2012) analysed 168 doubled haploid lines derived from a cross between two Chinese varieties, Haupei 3 and Yunai 57, to map QTL for numbers of grain per spike. Quantitative trait loci were observed on chromosomes 1A, 2B, 3B and 6A, with a major QTL on chromosome 1A accounting for 31.25% of the variance and another on chromosome 2B accounting for 46.75%, with a favourable allele contributed by Yumai 57 being associated with an increase of 5.69 kernels per spike.

Grain number was also the target of Zhang *et al.* (2012a), who used a Chinese wheat mini core collection to screen for alleles with a significant genetic effect on grain number [a mini core collection is a subset of a collection of genotypes (germplasm) in which all or most of the genetic diversity in the full collection is represented (Upadhyaya *et al.*, 2010)]. Association analyses identified 23 loci with a strong, positive effect, indicating that there is still considerable genetic potential for increasing grain number and therefore yield in Chinese wheat.

Li *et al.* (2012) looked for QTL controlling grain weight, but while loci were identified on chromosomes 1B, 2A, 2D, 4A, 4B and 7B, no single QTL was found to be effective at all stages of grain growth. Many QTL interacted with one or more other QTL, suggesting that the genetic architecture underlying the control of grain weight involves a collection of genes with additive and epistatic effects.

The relationship between grain size and shape was studied in multiple mapping populations, elite varieties and a broad collection of ancestral wheat species by Gegas *et al.* (2010). The study found grain size and shape to be largely independent traits under the control of a limited number of discrete genetic components. Distinct QTL were identified on chromosomes 1A, 3A, 4B, 5A and 6A.

The cloning of a major QTL for rice grain number (Ashikari *et al.*, 2005) illustrates how these studies can lead to the identification of candidate genes and markers for use in breeding programmes. Gn1a (grain number 1a) encodes a cytokinin oxidase/dehydrogenase (CKX), and when the expression of this gene is reduced, cytokinin accumulates in inflorescence meristems, thereby increasing their activity. This results in an increased number of reproductive organs and ultimately an enhanced number of grains. Impaired CKX function in Arabidopsis has similar effects on meristem size and activity, resulting in an increase in seed yield of over 50% (Bartrina *et al.*, 2011) and in wheat, TaCKX6, an orthologue of the rice gene, has since been found to be associated with 1000 grain weight (Zhang *et al.*, 2012b).

In conclusion to this section, it is clear that the genetics underpinning wheat grain yield is complex, with QTL scattered across almost all of the chromosomes (Table 4). Each QTL could contain many genes, but as the density of marker coverage improves it may be possible to narrow these QTL until it becomes feasible to characterise the individual genes contained within them. The identification of a cytokinin oxidase/dehydrogenase gene as one that potentially plays a role in the control of 1000 grain weight shows how the characterisation of QTL can lead to the development of molecular tools for use in breeding programmes. The other conclusion that emerges from these studies is that chromosome 3B was shown to carry important QTL in three independent studies involving different mapping populations (Cuthbert *et al.*, 2008; Bennett *et al.*, 2012; Yuan *et al.*, 2012) and this warrants further investigation.

**Table 4:** Quantitative trait loci (QTL) affecting yield and yield-related traits in wheat

Yield factor	QTL/potential QTL	References
1000 grain weight	Cytokinin oxidase/dehydrogenase Major QTL affecting grain number in rice; 7D	Zhang *et al.*, 2012b Ding *et al.*, 2011
		
Harvest index	1A, 2D, 3B and 5A	Cuthbert *et al.*, 2008
Average seed weight per spike	1B, 2B, 2D, 5A and 6B;	Wu *et al.*, 2012
	7D	Ding *et al.*, 2011
	3B	Bennett *et al.*, 2012
Canopy temperature and grain yield	3BL largest effect under heat stress conditions with RAC875 allele increasing grain yield by 131 kg ha^−1^	
Grain number per spike	1A, 2B, 3B, and 6A 5D close to vernalization gene *Vrn-D1*	Yuan *et al.*, 2012 Ding *et al.*, 2011
Yield	3A, 3D, 4D, 5B and 7A	Bennett *et al.*, 2012
Grain number	1B, 2A, 2D, 4A, 4B and 7B	Li *et al.*, 2012

## Synthetic hybrids

As described in the Introduction of this article, hexaploid bread wheat arose as the result of a rare hybridisation event that occurred only 10 000 years or so ago between tetraploid wheat and a diploid wild relative, *T. tauschii* (goat grass; also called *A. tauschii* and *A. squarosa*). This presumably occurred only once, and all commercial hexaploid wheat varieties must be descended from that single, recent event, meaning that there is limited genetic diversity in the hexaploid wheat gene pool. As a result, there is considerable interest in the much greater genetic diversity that must be present in wheat’s wild relatives and the introgression of useful traits into breeding programmes (Maxted & Kell, 2009). Indeed, there are many different genotypes within the wild *T. tauschii* population, and synthetic hexaploids have been created by hybridising tetraploid *Triticum durum* with some of these diverse genotypes. Fertile offspring are derived by artificially inducing chromosome doubling.

The use of synthetic hybrids was pioneered by CIMMYT (Dreisigacker *et al.*, 2008), which has produced over 1000 different synthetic wheat hybrids in this way. Many of these synthetic wheats have shown good disease resistance and tolerance of abiotic stresses, including drought stress (Rattey & Shorter, 2010). Four cultivars derived from CIMMYT synthetic hexaploids are now grown in China and Chinese researchers have recently made similar synthetic lines themselves (Yang *et al.*, 2009). Ten CIMMYT synthetics have also been backcrossed with local cultivars in the southern USA and the populations derived from these crosses showed improved yield in locations where disease pressure was high (Cooper *et al.*, 2012).

## The role of biotechnology

In any review of the prospects of increasing crop yield it is impossible to ignore the potential role of biotechnology. Currently that means genetic modification (GM), but it could soon mean the application of one or more of what might be called ‘post-GM’ technologies (Halford, 2012). These include cisgenesis (also known as intragenesis), which is GM using DNA entirely from the same species as the host plant; zinc finger nuclease technology, which uses artificial enzymes produced by fusing a zinc finger binding domain to the non-specific DNA cleavage domain of a restriction enzyme to introduce highly specific, targeted alterations to the DNA sequence; oligonucleotide-directed mutagenesis, which is based on site-specific mutation of a target gene by the introduction of an oligonucleotide; and RNA-dependent DNA methylation, which uses double-stranded RNAs to induce cytosine methylation of DNA, leading to the formation of transcriptionally silent heterochromatin.

Genetic modification is not new, of course, and there are successful, commercial GM traits for herbicide tolerance, insect resistance, virus resistance, shelf-life and oil composition in a variety of crop species (Halford, 2012). The concern for wheat breeding is that it is not one of the crop species for which there is an established biotech market, and with much of the investment in biotech happening in the USA, where wheat is a relatively low-value crop compared with maize and soybean, and Asia, where wheat ranks behind maize and rice in importance, wheat biotech is years behind that of other crops, particularly maize and soybean. Even Monsanto, the company that is generally so bullish about the potential of crop biotechnology, pulled out of wheat biotech in 2004 because of opposition from some US wheat farmers to the introduction of glyphosate-tolerant wheat. Monsanto has since returned to wheat, but it is developing new traits and any commercial release is years away.

**Figure 4:**
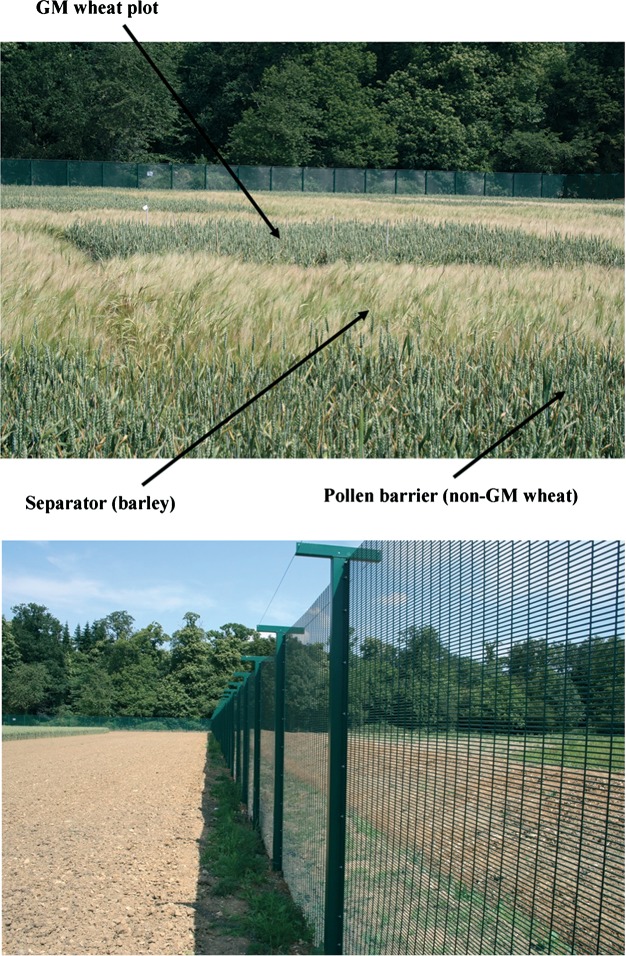
Field trial of genetically modified wheat in the United Kingdom, 2012. Top: view showing small plot surrounded by ‘separator’ and ‘pollen barrier’ plants. Bottom: Hi-tech security fencing and anti-ramming ditch to prevent unauthorised access to the site.

The region where most investment in wheat biotech might have occurred is Europe, where wheat is the major crop. However, genetic modification of crops remains such a contentious and over-regulated technology in Europe that there is no prospect at all of significant investment in the development of any GM crop for the European market. In the UK, for example, there was not a single field trial of GM wheat between 2002 and 2012, when a small trial was conducted to assess aphid, predator and parasitoid behaviour in GM wheat producing an aphid alarm pheromone (Beale *et al.*, 2006). Images of the trial are shown in Fig. 4 and two things stand out, particularly when considered in the context of the 160 million ha of GM crops being grown worldwide in 2012 (James, 2012). First, despite the fact that wheat is almost entirely self-pollinating and has no wild relatives in the UK, the tiny plots of GM wheat had to be surrounded by a ‘separator’ crop of barley and a ‘pollen barrier’ of non-GM wheat in order to satisfy regulators. Second, the trial had to be protected behind security fencing with a 24-h security presence to prevent vandalism.

There are some indications that rising food prices are changing the views of the UK public on crop biotechnology. Research carried out on behalf of the Crop Protection Association in 2013, for example, found that 35% of consumers would support GM foods being stocked on supermarket shelves (Crop Protection Association, 2013). This rose to 44% if the technology kept food prices down: still a minority, but a significantly larger one than has been recorded before. Nevertheless, there is no prospect of a GM wheat variety being launched in the UK or anywhere else in Europe and while that remains the case companies are likely to remain reluctant to invest in the genetic modification of wheat for increased yield or anything else.

## Anticipating and responding to climate change

If the predictions of climate change are correct, global warming will cause changes in temperature at a rate unmatched by any temperature change over the last 50 million years. For example, the temperature changes that have occurred between ice ages and warm interglacial periods during the last million years were of 4–7°C, but they occurred relatively gradually, with the global warming at the end of each ice age taking approximately 5000 years. The current rate of global climate change is much more rapid. The upper end of the range predicted by Global Climate Models in the International Panel for Climate Change (IPCC) Fourth Assessment Report is a 5°C increase in global mean temperature by the end of this century (Solomon *et al.*, 2007), and this prediction is repeated in the draft Fifth Assessment Report (http://www.ipcc.ch/report/ar5/wg1/). The potential effects of such an increase on crop yields, earnings, reliability of supply, food quality and food safety have been reviewed in detail by Vermeulen *et al.* (2012).

Climate change is predicted to bring about an increase in the frequency and severity of extreme weather events, similar to the droughts experienced in Australia and Russia in the last decade (Fig. 3). In both those cases, lack of rain was accompanied by high temperature and both stresses would likely have affected grain yield. Drought stress can be devastating at any time during wheat development, while increased temperature shortens the growing period, reducing yield. Heat stress at flowering is especially damaging, resulting in much lower grain number and substantial yield losses (Mitchell *et al.*, 1993). Heat and drought stress provoke different responses in plants but the dividing line is often blurred as one exacerbates the effects of the other. While both are already a problem, the range over which they impact seriously on crop yields and the frequency with which they do so are predicted to increase as a result of global warming.

The fact that plant breeders will have to develop new varieties for climatic conditions that do not currently prevail makes crop simulation modelling particularly important (reviewed by Semenov & Halford, 2009). The aim of this modelling is to enable a broad understanding of complex processes that would not be possible by studying single or small groups of genes responding, for example, to one particular environmental stimulus. Mathematical equations are developed from the analysis of large datasets from transcriptomic, proteomic and metabolomic analyses. Experiments are then carried out to validate and, if necessary, refine the model. In the case of climate change, crop simulation modelling is used in conjunction with modelling of future climates.

Climate scenarios predict warmer, drier summers for the UK by 2050, but wetter winters (Semenov, 2007), so UK wheat could benefit from more water in the soil in the early part of the season but be faced with twin challenges of drought and heat in the summer. Semenov *et al.* (2009) used crop simulation modelling to compare the effects of these two stresses on UK wheat yield. They concluded that the probability of maximum temperature exceeding 27°C around flowering would increase significantly but that this would be offset by wheat phenology (the rate of wheat growth and development is temperature-dependent), so flowering would occur earlier in the season. This would also mean that the crop would avoid the most severe summer drought stress by maturing earlier. Overall, the impact of drought stress on wheat in the UK was predicted to decrease with climate change, so that an increase in the frequency of heat stress around flowering actually represented a greater risk (Semenov & Halford, 2009; Semenov *et al.*, 2009). This was consistent with the conclusions of a series of studies in the 1990s that predicted severe effects of temperature increases on wheat yield (Wheeler *et al.*, 1996, 2000; Ferris *et al.*, 1998). However, some studies have suggested that the impact of drought on UK wheat yield will increase with climate change and that this should be the main focus for research (Foulkes *et al.*, 2007; Witcombe *et al.*, 2008), while extremes of both heat and drought have been predicted to cause an increase in wheat crop failure rates in Northeast China (Challinor *et al.*, 2010).

All these predictions are based on a number of assumptions and the effects vary from one location to another and are variety-dependent. In our view, it would be premature to discount the potential yield losses from either stress and both drought and heat tolerance are important targets. However, in addition to heat and drought, plants will have to cope with a third problem, that of steeply rising atmospheric CO_2_ concentration. Plants require CO_2_ to photosynthesise, of course, and a modest increase in atmospheric CO_2_ levels would almost certainly enhance crop productivity if it were not accompanied by heat and drought stress. Indeed, photosynthesis evolved when CO_2_ levels were many times higher than they are today and the long-term decline in CO_2_ will eventually result in its concentration falling below the point where plants can photosynthesise efficiently. There have been blips in this decline in the past, probably as a result of periods of unusual volcanic activity, such as during the ‘mid-Cretaceous superplume’ (Caldeira & Rampino, 1991). Currently, the planet is on the up-curve of another blip, this time resulting from human activity, and it is the effect of this blip over the coming decades and centuries that is the more immediate threat. The atmospheric CO_2_ concentration has risen from a pre-industrial level of 270 parts per million (ppm) to 390 ppm and is rising at 1–2 ppm per year, taking it to levels not seen for 20 million years.

Several studies have been carried out on the long-term acclimation of crop plants to elevated CO_2_ using free-air CO_2_ enrichment (FACE), in which CO_2_-enriched air is passed over plants in an experimental plot from a system of pipes. Ewert et al. (2002) used crop simulation models to predict the effects of elevated CO_2_ concentration on wheat yield, using data from FACE experiments and open-top chamber experiments in Germany. They concluded that crop simulation models could be used to predict wheat growth and yield for different CO_2_ and drought treatments in a field environment, but that there was still uncertainty about the combined effects of CO_2_ concentration and drought that required further testing and enhancement of the model.

Jamieson *et al.* (2000) also tested three crop simulation models against data from FACE wheat experiments, but in this case included results of experiments in which the amount of applied nitrogen as well as atmospheric CO_2_ concentration was varied. The models all predicted yield trends in terms of green area index, biomass accumulation and yield. The study showed that changes in CO_2_ concentration affected light use efficiency, whereas nitrogen application affected the green area index.

Oleson *et al.* (2007) predicted some positive consequences of climate change for European agriculture when the impact of increased CO_2_ concentration was included, but with considerable uncertainty arising from emission scenarios, the climate and crop simulation models that were used, and local soil and climatic conditions. The study showed large increases in yield of winter wheat for northern Europe, but smaller increases or even decreases in southern Europe. It also predicted that the area of maize cultivation in Europe would expand by 30–50% by the end of the century. Overall, the study foresaw increases of 35–54% in net primary productivity in northern Europe as a result of a longer growing season and higher CO_2_ concentrations.

Richter and Semenov (2005) also predicted mixed outcomes of climate change on wheat yield, using crop simulation modelling to evaluate changes in drought indicators and yield of winter wheat in the UK, comparing climate scenarios constructed for the 2020s and 2050s with a 1960–1990 baseline. Soil moisture deficit and potential yield loss due to drought were predicted to increase, especially on shallow soils, but this would be offset by a CO_2_-related increase in radiation use efficiency. The net effect would be an increase in average wheat yields of 15–23% by the middle of the century.

The results of the FACE studies have been reviewed by Leakey *et al.* (2009), who listed a number of major conclusions. On the positive side, elevated CO_2_ stimulates photosynthetic carbon gain and net primary production, while nitrogen use efficiency is improved and water use is decreased. However, on the negative side, the activity of ribulose 1,5-bisphosphate carboxylase/oxygenase (Rubisco), which catalyses carboxylation of ribulose-1,5-bisphosphate, the first step in carbon fixation, is down-regulated and photosynthesis does not increase as much as might otherwise be predicted (Long *et al.*, 2006). Dark respiration is also stimulated, and a decreased proportion of photosynthate is partitioned to the harvested organs. Overall, yield is increased but by less than might be expected. Understanding why this is and how it can be remedied is an important challenge.

Rubisco and photosystem II (the protein complex that captures light energy and initiates the electron transport chain in the first stage of photosynthesis) are also inhibited by heat (Al-Khatib & Paulsen, 1999). The reduction in Rubisco activity occurs because the enzyme responsible for maintaining its activity, Rubisco activase, is labile at even moderately high temperatures. Genetic manipulation of Rubisco activase to improve its stability at high temperatures improves photosynthetic efficiency under moderate heat stress in Arabidopsis and is a potential target for crop biotechnology (Kurek *et al.*, 2007).

So far, we have considered what stresses plants might have to cope with as a result of climate change, but there is another angle to the climate change issue in that governments around the world are committed to reducing the emissions of greenhouse gases that are believed to be responsible for global warming and agriculture accounts for a significant proportion of these emissions. In the UK, for example, the Climate Change Act (2008) commits the country to an 80% reduction in greenhouse gas emissions by 2050 across all sectors. The UK government calculates that agriculture is responsible for about 9% of total UK emissions (Economics Group, Defra, 2011), with 55% coming from nitrous oxide (N_2_O), 36% from methane and 9% from CO_2_, and there is no indication that agriculture will be exempted from making its contribution to meeting the government’s target. Note that the figure of 9% may be typical of a developed country but that globally the contribution of agriculture to greenhouse gas emissions may be as high as 30% (Vermeulen *et al.*, 2012; Tubiello *et al.*, 2013).

Most of the N_2_O that is emitted from UK agricultural systems arises from the application of synthetic fertiliser to arable soils (Economics Group, Defra, 2011). Clearly this will have to change if the climate change commitments are to be met, but fertiliser production is becoming an increasingly expensive process anyway as the price of oil increases, something that is unlikely to be reversed with the approach of peak oil (the point when the maximum rate of global petroleum extraction is reached, after which the rate of production enters terminal decline).

## The importance of considering food safety

The most commonly used definition of food security comes from the UN’s FAO: ‘Food security exists when all people, at all times, have physical, social and economic access to sufficient, safe and nutritious food to meet their dietary needs and food preferences for an active and healthy life’. In other words, food has to be safe to eat and nutritious as well as available. Western consumers might put it more strongly, with several decades of plentiful food leading to food safety rising above availability as a concern. Plant breeders must be aware of this, not only because consumer safety must be a priority, but also because the food industry is extremely sensitive to the risk of bad publicity for its products and companies will act swiftly if switching to a different raw material or supplier reduces the likelihood of a product attracting damaging headlines.

The formation of the processing contaminant acrylamide during the high-temperature cooking and processing of foods made from potatoes, cereals and other crops is an example of just such a food safety problem, and one that the food industry would definitely not welcome being made any worse. Acrylamide, which was discovered in a range of mainly plant-derived foods in 2002 (Tareke *et al.*, 2002), is classified by the World Health Organisation and the International Agency for Research on Cancer as a Group 2a carcinogen (‘probably carcinogenic to humans’) (International Agency for Research on Cancer, 1994) and it also has effects on neurological and reproductive systems at high doses (Friedman, 2003).

Acrylamide forms as a result of the Maillard reaction, an umbrella name given to a series of non-enzymic reactions between sugars and amino groups, principally those of amino acids, that occur during high-temperature cooking and processing, including frying, baking and roasting. Detailed descriptions of the Maillard reaction have been provided previously (Nursten, 2005; Mottram, 2007; Halford *et al.*, 2011). It was an important reaction for the food industry long before acrylamide was discovered to be one of its products because it also produces the melanoidin compounds that give fried, roasted and baked products their colour, and a host of other compounds that impart aroma and flavour (Mottram, 2007; Halford *et al.*, 2011). This makes the situation more difficult for the food industry because these are the compounds that give foods the flavours, aromas, textures and colours that define brands and are demanded by consumers.

The Maillard reaction requires a reducing sugar such as glucose, fructose or maltose, although sucrose will participate if it is first hydrolysed through enzymatic, thermal or acid-catalysed reaction (De Vleeschouwer *et al.*, 2009). The first step is the condensation of the carbonyl (C=O) group of the reducing sugar with an amino group. This produces a Schiff base, which cyclises and then undergoes rearrangement, enolisation, deamination, dehydration and fragmentation to give rise to sugar dehydration and fragmentation products containing one or more carbonyl groups. These carbonyl compounds react with amino groups and other components, resulting in the formation of a plethora of different compounds. One important reaction is Strecker degradation, whereby an amino acid is deaminated and decarboxylated to give an aldehyde, and a Strecker-type reaction involving asparagine is the major route for acrylamide formation (Mottram *et al.*, 2002; Stadler *et al.*, 2002; Zyzak *et al.*, 2012).

The FAO/WHO Joint Expert Committee of Food Additives (JECFA) and other risk assessment bodies have recommended that acrylamide levels in food be reduced as a matter of priority (Joint FAO/WHO Expert Committee of Food Additives, 2011) and the European Commission issued ‘indicative’ levels for acrylamide in food in early 2011 (European Commission, 2011). Indicative values are not safety thresholds, but are intended to indicate the need for an investigation into why the level has been exceeded. Nevertheless, products that have been found to exceed the indicative value have attracted damaging publicity.

Wheat products are some of the most important contributors to dietary acrylamide intake across Europe, (European Food Safety Authority, 2011) along with potato products and coffee. Manufacturers have devised many strategies for reducing acrylamide formation by modifying food processing and these have been compiled in a ‘Toolbox’ produced by FoodDrinkEurope (2011). Further progress may be possible by variety selection and optimising crop management (reviewed by Halford *et al.*, 2012). The major determinant of acrylamide-forming potential in wheat flour is the concentration of free asparagine (Muttucumaru *et al.*, 2006; Granvogl *et al.*, 2007; Curtis *et al.*, 2009) and the same is true for rye (Curtis *et al.*, 2010; Postles *et al.*, 2013) and probably for other cereals. Reducing the free asparagine concentration of wheat grain is therefore a priority and there should be no doubt that food manufacturers expect plant breeders to engage on the acrylamide issue.

Breeders must also beware of inadvertently increasing acrylamide-forming potential while selecting for yield or other traits, because there is evidence from several crop species of links between asparagine metabolism, yield and protein content. For example, simultaneous silencing of asparagine synthetase genes *AST1* and *AST2* in potato tubers, which was done in an effort to reduce acrylamide-forming potential, resulted in small tubers in field-grown potatoes (Chawla *et al.*, 2012). This problem was overcome by silencing of *AST1* on its own, which reduced asparagine levels in tubers without reducing tuber size, probably because *AST1* controls asparagine levels in tubers while *AST2* has a broader role, but the link between asparagine metabolism and yield is clear. A different study, this time on soybean, showed a positive correlation between free asparagine levels in developing seeds and protein concentration at maturity (Pandurangan *et al.*, 2012).

Gaufichon *et al.* (2010) listed the biological functions of asparagine synthetase in plants as: nitrogen mobilization during seed germination; metabolic nitrogen recycling and flow in vegetative organs; nitrogen remobilization from source organs to sink organs and seed nitrogen filling and maturation. Lea *et al.* (2007) reviewed asparagine in plants more broadly, highlighting the accumulation of free asparagine during normal physiological processes such as seed germination and nitrogen transport, but also in response to a variety of stresses, including mineral deficiencies, drought, salt, toxic metals and pathogen attack. The authors concluded that there is now clear evidence that free asparagine accumulates in plants during periods of low rates of protein synthesis combined with a plentiful supply of reduced nitrogen. These studies show that modification of wheat physiology to improve yield could inadvertently affect asparagine concentration in the grain, and this is something that breeders must be aware of.

Acrylamide is not the only processing contaminant causing concern. Furan (a heterocyclic compound consisting of a five-membered aromatic ring comprising four carbon atoms and one oxygen) and more complex compounds containing furan rings are also receiving attention. Furan causes liver cancer in rodents (Leopardi *et al.*, 2010) and is classified by the International Agency for Research on Cancer as ‘possibly carcinogenic’ to humans (Group 2B) (International Agency for Research on Cancer, 1995).

Like acrylamide, furan forms through the Maillard reaction, but it also arises from the thermal degradation of polyunsaturated fatty acids, directly from sugar breakdown and from ascorbic acid (Perez Locas & Yaylayan, 2004; Crews & Castle, 2007; Moro *et al.*, 2012). A compound that has attracted some attention in bakery products is hydroxymethylfurfural (HMF) (Ramírez-Jiménez *et al.*, 2000), which consists of a furan ring with both aldehyde and alcohol functional groups. One of the metabolic products of HMF is 5-sulfoxymethylfurfural, which can form adducts with DNA or proteins and has indicated potential toxicity and carcinogenicity in rodent toxicology studies (Husøy *et al.*, 2008).

It is important that breeders take on board the need to consider the potential for processing contaminant formation when breeding for yield or other traits. Contaminants arise from other sources, of course, with mycotoxins produced by fungal activity being an obvious example. However, it is highly unlikely that improvements in yield would inadvertently bring about an increase in fungal infection and mycotoxin contamination.

## Conclusions

At the turn of the 21st century, it was a popular view that food security in Europe would never be a problem, and that food shortages elsewhere resulted from problems in distribution rather than production. Indeed, the European Union’s Common Agricultural Policy was regarded by many to be encouraging farmers to over-produce food that there was no demand for. A few commentators pointed out that the food surpluses that were being generated even in Europe were actually quite small as a percentage of the total and that it was much better to be in a position of surplus rather than deficit, but they were largely ignored.

The debate changed very rapidly during the first decade of the century, as increasing demand coupled with extreme weather events in major crop-exporting countries drove up prices and eroded reserves. Food security is now at the top of the agenda and improving crop yield is seen as an important part of a strategy to prevent food shortages and the political and economic upheaval that would be likely to accompany them.

Wheat breeding has already been extremely successful in increasing yield in some regions, with the UK, as we have described, being one example (Fig. 1). This could, of course, mean that further improvement will be difficult to achieve. Perhaps addressing the problems of those areas of the world where yield is still much lower (Table 1) should therefore be a priority. However, if yield were increased elsewhere, farmers in the UK and other developed countries with high operating costs would need to see improvement as well in order to remain competitive. Furthermore, the high yield obtained in the UK and other very productive regions results not only from genotypic improvement but also from mechanisation and the application of large amounts of nitrogen fertilizer (typically 160–280 kg N ha^−1^ (Defra, 2010)) and other agrochemicals. This level of intensity is heavily dependent on fossil fuels and may not be sustainable.

The challenges facing global agriculture led Sir John Beddington, a former Chief Scientific Advisor to the UK government, to make the statement that ‘The challenge for global agriculture is to grow more food on not much more land, using less water, fertiliser and pesticides than we have historically done’. We concur with this view but reiterate that food safety is also important and the food industry would not thank breeders for high-yielding wheat varieties that have an increased potential for the formation of processing contaminants.
